# Feto-Maternal Effects of Adding Rifampicin to Ursodeoxycholic Acid in the Treatment of Intrahepatic Cholestasis of Pregnancy

**DOI:** 10.7759/cureus.32509

**Published:** 2022-12-14

**Authors:** Anjali Kumari, Avinash Kumar, Manoj Kumar, Swati Swati

**Affiliations:** 1 Department of Obstetrics and Gynecology, Anugrah Narayan Magadh Medical College, Gaya, IND; 2 Department of General Medicine, All India Institute of Medical Sciences, Patna, IND; 3 Department of Pediatrics, All India Institute of Medical Sciences, Deoghar, IND; 4 Department of Obstetrics and Gynecology, All India Institute of Medical Sciences, Deoghar, IND

**Keywords:** obstetric cholestasis, feto-maternal outcome, neonatal outcome, rifampicin, ursodeoxycholic acid, intrahepatic cholestasis of pregnancy

## Abstract

Background

Various pharmacological agents are used to manage intrahepatic cholestasis of pregnancy (ICP) for maternal pruritus and to lower serum bile acids in fear of adverse fetal outcomes. Ursodeoxycholic acid (UDCA) is the most widely used drug, but some patients do not respond to it. Neither UDCA nor any other drug being used for ICP is based on a high level of evidence.

Methods

A total of 108 pregnant women with ICP who were receiving UDCA with or without rifampicin were included in a prospective observational study from December 2018 to November 2020. Seventy-eight patients receiving UDCA only were labeled as group A, and 30 patients receiving UDCA with rifampicin were labeled as group B.

Results

The study subjects were comparable in both groups with respect to demographic factors. Pruritus, being the major symptom of ICP, has a mean (standard deviation (SD)) onset at 30.02 (2.93) weeks and 26.70 (4.56) weeks of gestation in groups A and B, respectively. Group B patients had earlier onset of symptoms and earlier mean (SD) gestational age at diagnosis at 28.89 (4.29) weeks compared to 32.47 (2.93) weeks in group A. Therefore, the mean (SD) gestational age to start UDCA was early in group B (29.32 (4.24) weeks). Relief in itch from UDCA was seen in 93.59% (73) in group A and 10% (3) in group B (partial relief). The mean (SD) duration for receiving only UDCA was 3.84 (2.07) weeks and 2.86 (1.58) weeks, respectively, for groups A and B. The mean (SD) gestational age at starting rifampicin was 32.11 (3.4) weeks for group B (n = 30). UDCA plus rifampicin was given for a mean (SD) duration of 3.48 (1.42) weeks. The mean (SD) dosage of UDCA given per day was 911.54 (229.05) mg in group A and 880 (260.50) mg in group B (p value = 0.563). The mean (SD) dosage of rifampicin used in group B was 700 (363.89) mg/day. The mean (SD) of baseline bile acids (pretreatment) was 36.94 (13) umol/L and 42.50 (15.23) umol/L in groups A and B, respectively (p value = 0.274). At the two-week follow-up, the mean (SD) value of serum bile acids was 22.92 (10.67) umol/L and 14.88 (10.27) umol/L in groups A and B, respectively (p value = 0.039). Group B having an earlier onset of ICP also had early gestational age at delivery at 35.70 (2.57) weeks versus 37.011 (1.18) weeks in group A. Of the babies in groups A and B, 63% and 50% were born full term, respectively. There was no significant difference in the mode of delivery for both study groups. The mean (SD) birth weight of babies was 2,706.85 (206.19) grams for group A and 2,522.67 (342.20) grams in group B. Adverse neonatal outcomes for both groups were comparable (68.5% in group A and 70% in group B) (p value = 0.881). Of the patients, 9% and 6.7% had antepartum stillbirth in groups A and B, respectively. Of the babies in groups A and B, 10.3% and 6.7% were born with dark-colored meconium or placental membranes and cord stained with meconium, respectively. In groups A and B, 9% and 6.7% of the babies were born with thin/light green meconium-stained liquor, respectively.

Conclusion

Rifampicin, if added to UDCA for the management of ICP, does not cause any adverse fetal outcome. It is a useful adjunct to UDCA for severe and/or resistant ICP, and it helps improve pruritus and serum bile acids.

## Introduction

It is well known that the metabolic, synthetic, and excretory functions of the liver are affected by high levels of serum estrogen and progesterone during pregnancy. Liver diseases complicating pregnancy have been seen to resolve either spontaneously or following delivery. Intrahepatic cholestasis of pregnancy (ICP) is a pregnancy-specific liver disease that may lead to adverse fetal outcomes including preterm delivery, meconium staining of the amniotic fluid, and stillbirth [1,2]. ICP, also known as obstetric cholestasis (OC), usually occurs in late pregnancy.

It has a variable worldwide prevalence ranging approximately between 0.3% and 5.6% of pregnancies [3-5]. Approximately 1% of all pregnancy in the United States is affected by ICP [6]. Due to genetic background, family clustering, and demographic variations, the highest incidences have been reported from Chile-Bolivia (6%-27%) [7]. In the United Kingdom, it affects approximately 0.7% of pregnant women, equating to approximately 6,000 women per year [8]. In women of Indian-Asian or Pakistani-Asian origin, its incidence is 15 in 1,000 (1.5%) [9]. In Bihar, the incidence at tertiary care centers such as Darbhanga Medical College, Bihar, is approximately five cases in 1,000 pregnancies.

Typically, obstetric cholestasis presents after 28 weeks of gestation and usually progresses until delivery. It is more common in women with multifetal pregnancy, following in vitro fertilization, in older women, more frequent after 35 years of age (25% of cases), in those with a history of gallstones or hepatitis C infection, and in sisters of affected women [10].

Pruritus is the most characteristic manifestation, and it tends to show a predilection to palms and soles. It is intense and persistent, which worsens at night. There are no constitutional symptoms as seen with other liver diseases. Pruritus usually disappears within 24-48 hours after delivery, but biochemical and histological abnormalities have been found to resolve after a few weeks to months. Rarely, symptoms may persist for several weeks postpartum. ICP is seen to recur in 60%-70% of subsequent pregnancies [8].

Pruritus is often seen to precede biochemical abnormalities. Serum bile acids are raised and are considered the most sensitive and specific markers of ICP. The higher the levels of serum bile acids, the more the chances of adverse fetal and perinatal outcomes. This test may not be available widely at all centers [8]. Apart from serum bile acids, other liver function tests are altered, such as alanine transaminase (ALT), which is more sensitive for ICP than aspartate transaminase (AST) and serum bilirubin. ALT and AST may rise two- to 10-fold. Bilirubin is almost normal in the majority, but if raised, it tends to be conjugated hyperbilirubinemia. Rarely, patients develop clinical jaundice. The Royal College of Obstetricians and Gynaecologists recommends that in the absence of a serum bile acid test, ICP may be diagnosed in pregnant women presenting with pruritus and abnormal liver function tests whose other causes of pruritus have been excluded. However, it can lead to overdiagnosis, overtreatment, and unnecessary interventions [11].

Various pharmacological agents belonging to different classes are used to manage ICP. These are ursodeoxycholic acid (UDCA), cholestyramine, dexamethasone, S-adenosylmethionine, and, recently, rifampicin. None of these is based on high-level evidence, and also, there are various concerns over costs, side effects, and possible harms. The pharmacological treatment of ICP is the subject of a 2020 systematic review in the Cochrane Library [12]. Ursodeoxycholic acid (UDCA) is widely used for the same. Three weeks of UDCA treatment is known to improve some biochemical markers of ICP irrespective of disease severity. Significant relief from pruritus and marked reduction of serum bile acids have been found only in patients having severe ICP [13].

Rifampicin has been used in severe cholestatic liver diseases, and its mechanism of action is not very well understood. In patients with primary biliary cirrhosis (PBC), it has been shown to reduce serum bilirubin, enhance hepatic efflux of organic anions including serum bile acids, and improve pruritus. The mechanism of action in such diseases is complementary to those of UDCA. Combination therapy with rifampicin and UDCA might therefore be more effective than UDCA treatment alone [14,15]. For the safety profile of rifampicin, it is very well known that rifampicin has been used to treat tuberculosis in pregnant women. Rifampicin has no known teratogenic effects, although an effect on vitamin K metabolism mandates parenteral neonatal administration of vitamin K [16].

There are only a few studies regarding combining both these drugs in obstetric cholestasis. This paper presents an observational study from a tertiary care teaching hospital in Bihar, India, on the use of rifampicin in addition to UDCA in pregnant women with ICP.

The research question was “Is there any benefit of adding rifampicin to UDCA in those patients with ICP who do not or partially respond to UDCA?” We aim to study the feto-maternal effects of adding rifampicin to UDCA for the treatment of those patients with ICP who have no/partial relief from UDCA, and our objective(s) are as follows: to estimate what percentage of patients get relief in itch after adding rifampicin to UDCA, to estimate the dosage of rifampicin needed in those subsets of ICP patients who need it in addition to UDCA, and to observe if the addition of rifampicin harms the neonatal outcome in comparison to that for UDCA alone.

## Materials and methods

This study was a prospective observational study, which was carried out in the Department of Obstetrics and Gynecology, Darbhanga Medical College and Hospital, Laheriasarai, Bihar (approval number: IRC/41/2018). A total of 108 pregnant women were included in the study over a period of two years from December 2018 to November 2020.

Inclusion criteria

The inclusion criteria for the study are as follows: singleton pregnancy and pregnancy with itching plus raised serum bile acid (the normal value of fasting serum bile acids is ≤10 umol/L, and levels more than 10 umol/L is ICP and above 40 umol/L is severe ICP), and/or moderately raised AST/ALT (the normal value of fasting serum bile acids is ≤ 10 umol/L, and levels more than 10 umol/L is ICP and above 40 umol/L is severe ICP) (moderately raised AST/ALT is <3 times the upper range of normal value (35 × 3 = 105 U/L)), and/or mildly raised serum bilirubin (up to 4mg%), and/or urine bile salt positive. All patients were not sampled for serum bile acids (the reasons for this are coming in an emergency with a non-fasting condition, in labor/fetal distress, etc.), so for a quick diagnosis of ICP, these subsets of study subjects were tested for urine bile salt/pigments.

Exclusion criteria

The exclusion criteria are as follows: multiple pregnancies, other causes of pruritus, and other causes of liver dysfunction (pregnancy-specific: preeclampsia, hemolysis, elevated liver enzymes, and low platelets (HELLP) syndrome, acute fatty liver of pregnancy, and hyperemesis gravidarum; non-pregnancy-associated: viral hepatitis, primary biliary cirrhosis, primary sclerosing cholangitis, autoimmune hepatitis, drug-induced liver injury, biliary obstruction, and veno-occlusive disease).

**Figure 1:**
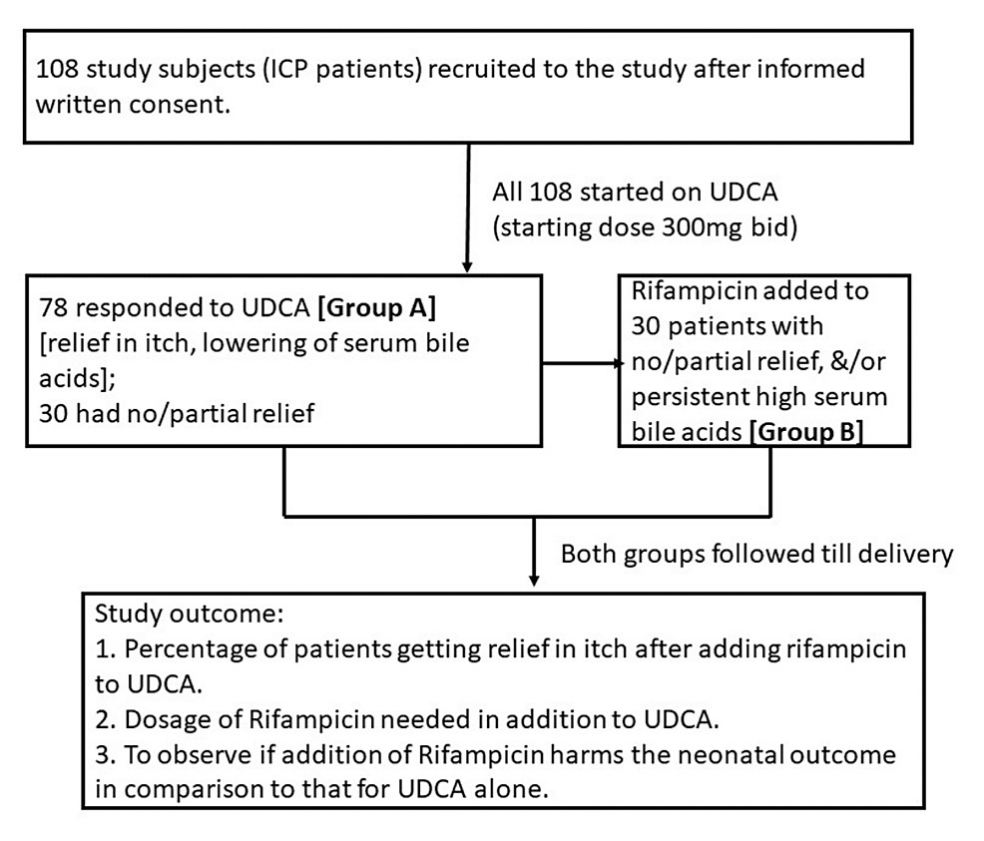
Flowchart showing study methodology in brief ICP: intrahepatic cholestasis of pregnancy, UDCA: ursodeoxycholic acid

All the 108 study subjects were started on UDCA, but 78 responded to UDCA and were labeled as group A. Rifampicin was added to the remaining 30 grouped in group B. Therefore, the study had two groups: group A (who received UDCA only) and group B (who received UDCA followed by rifampicin). Both groups were followed until the delivery of the baby, and data collection was done regarding drug treatment, symptom relief, serum biochemistry, and fetal-maternal outcomes (Figure 1).

Seventy-eight patients in group A, who were treated only with UDCA, were followed with repeat serum bile acid investigation after two weeks, and in 17 study subjects, predelivery serum bile acids were measured as they attended hospital in the late trimester (late here means 38 weeks plus, and these patients were immediately taken for delivery (induction of labor (IOL), augmentation of spontaneous labor, emergency cesarean (EMCS), and elective cesarean (ELCS))).

Group B consisted of 30 participants in whom rifampicin was added as an adjuvant drug to UDCA (for the following indications: elevated serum bile acid level, and/or there was no/partial relief in pruritus (no standard scale was used to measure relief of itch in ICP study subjects) (based on patient feedback in terms of satisfactory relief to UDCA, the decision of adding rifampicin was taken), and/or reduced fetal movement (in preterm) along with an assessment of fetal well-being).

Statistical analysis

Data were coded and recorded in a Microsoft Excel spreadsheet (Microsoft Corp., Redmond, WA, USA). Statistical Package for the Social Sciences (SPSS) version 23 (IBM SPSS Statistics, Armonk, NY, USA) was used for data analysis. Descriptive statistics were elaborated in the form of means/standard deviations (SDs) and medians/interquartile ranges (IQRs) for continuous variables, and frequencies and percentages for categorical variables. Group comparison for continuously distributed data was done using an independent sample t-test when comparing two groups. If data was found to be non-normally distributed, an appropriate non-parametric test in the form of the Wilcoxon rest was used. Linear correlation between two continuous variables was explored using Pearson’s correlation (if the data were normally distributed) and Spearman’s correlation (for non-normally distributed data). Statistical significance was kept at a p value of 0.05 with a 95% confidence interval (CI).

## Results

The mean (SD) age in group A was 26.81 (2.16) years and that in group B was 26.60 (3.42) years. The demographic profile of the study subjects is given in Table 1, which shows that the study subjects are comparable (p value > 0.05). For religion, more Hindu study subjects were seen as India and Bihar have Hinduism as a majority religion (p value < 0.05) [17].

**Table 1:** Demographic variables of the study subjects ***Significant at p < 0.05 ^1^t-test ^2^Fisher’s exact test ^3^Chi-squared test SD: standard deviation

Demographic parameters	Group A (n = 78)	Group B (n = 30)	p value
Mean ± SD age (years)	26.81 ± 2.16	26.60 ± 3.42	0.758^1^
Age group			0.250^2^
20-25 years	20 (25.6%)	12 (40%)	
26-30 years	54 (69.2%)	16 (53.3%)	
31-35 years	4 (5.1%)	2 (6.7%)	
Occupation			1.000^2^
Housewife	76 (97.4%)	29 (96.7%)	
Teacher	2 (2.6%)	1 (3.3%)	
Religion***			0.049^3^
Hindu	60 (76.9%)	28 (93.3%)	
Muslim	18 (23.1%)	2 (6.7%)	
Marital status (married)	78 (100%)	30 (100%)	1.000^3^
Socioeconomic status			0.215^3^
Low	53 (67.9%)	24 (80%)	
Middle	25 (32.1%)	6 (20%)	

The study subjects showed no significant difference with respect to obstetric and past medical/surgical history (Table 2). Group A comprised 50% primigravida and 50% multigravida. Group B had 46.7% primigravida and 53.3% multigravida. Of the study subject in groups A and B, 37.2% (29) and 36.7% (11) gave a history of itch and/or diagnosed ICP in previous pregnancies, respectively. In groups A and B, 9% (7) and 13.3% (4) have gallstones, respectively, and 3.8% (3) and 6.7% (2) had undergone cholecystectomy in the past, respectively.

**Table 2:** Obstetric and past medical/surgical history of the study subjects ***Significant at p < 0.05 ^1^t-test ^2^Fisher’s exact test ^3^Chi-squared test ICP: intrahepatic cholestasis of pregnancy, OC: obstetric cholestasis

Obstetric and medical history parameters	Group A (n = 78)	Group B (n = 30)	p value
Parity index			0.756^3^
Nullipara	39 (50%)	14 (46.7%)	
Primipara and multipara	39 (50%)	16 (53.3%)	
Tetanus immunization			0.941^3^
T1	28 (35.9%)	11 (36.7%)	
T1 and T2	50 (64.1%)	19 (63.3%)	
Previous number of antenatal checkups in the current pregnancy			0.222^2^
No visit	0 (0%)	1 (3.3%)	
1 visit	17 (21.8 %)	8 (26.7%)	
≥2 visits	61 (78.2%)	21 (70%)	
Past history			0.743^2^
Itching/ICP in the previous pregnancy	29 (37.2%)	11 (36.7%)	
Gallstone	7 (9%)	4 (13.3%)	
Cholecystectomy	3 (3.8%)	2 (6.7%)	
Nothing significant	39 (50%)	13 (43.3%)	
Past history of itch with combined OC pills	3 (3.9%)	2 (6.7%)	0.618^2^

Table 3 depicts the clinical and biochemical profiles of the study subjects. Pruritus being the major symptom of ICP had a mean (SD) onset at 30.02 (2.93) weeks of gestation in group A and 26.70 (4.56) weeks in group B (p value = 0.001, significant). The mean (SD) gestational age at clinical diagnosis (seeking consultation after the onset of pruritus and biochemical tests) for ICP was 32.47 (2.93) weeks in group A and 28.89 (4.29) weeks in group B (p value < 0.001). Mild ICP (serum bile acids < 40 umol/L) was present in 88.46% and 80% of the study subjects in groups A and B, respectively, whereas 11.54% in group A and 20% in group B had severe ICP (serum bile acids ≥ 40 umol/L) (p value = 0.06). The mean serum bile acid was 36.94 (13) and 42.5 (15.23) umol/L in groups A and B, respectively (p value = 0.274).

**Table 3:** Clinical and biochemical profile of the study subjects ***Significant at p < 0.05 ^1^t-test ^2^Fisher’s exact test ^3^Chi-squared test UDCA: ursodeoxycholic acid, SD: standard deviation, ICP: intrahepatic cholestasis of pregnancy, AST: aspartate transaminase, ALT: alanine transaminase, ALP: alkaline phosphatase

Clinical and biochemical parameters	Group A (n = 78)	Group B (n = 30)	p value
Mean ± SD gestation (weeks) at the onset of pruritus***	30.02 ± 2.93	26.70 ± 4.56	0.001^1^
Mean ± SD serum bile acids at diagnosis (umol/L)	36.94 ± 13.00	42.50 ± 15.23	0.274^1^
Mild ICP (serum bile acids < 40 umol/L)	69 (88.46%)	24 (80%)	0.062^3^
Severe ICP (serum bile acids ≥ 40 umol/L)	9 (11.54%)	6 (20%)	
Mean ± SD AST (IU/L) at diagnosis	74.14 ± 25.76	72.10 ± 24.99	0.708^1^
Mean ± SD ALT (IU/L) at diagnosis	79.17 ± 30.68	80.27 ± 30.73	0.868^1^
Mean ± SD ALP (IU/L) at diagnosis	133.14 ± 18.30	136.07 ± 21.29	0.510^1^
Mean ± SD serum bilirubin (mg/dL) at diagnosis	0.86 ± 0.22	0.79 ± 0.23	0.150^1^
Bile salt/pigment in urine at first clinical presentation	(n = 78)	(n = 30)	0.647^3^
Positive	43 (55.1%)	18 (60%)	
Not done	35 (44.9%)	12 (40%)	
Mean gestation at diagnosis (weeks)***	32.47 ± 2.93	28.89 ± 4.29	<0.001^1^
Mean gestation (weeks) at which UDCA started***	32.86 ± 2.99	29.32 ± 4.24	<0.001^1^
Relief of pruritus after UDCA	(n = 78)	(n = 30)	<0.001^3^
Yes***	78 (100%)	3 (10%)	
Time (in weeks) for relief of pruritus with UDCA***	(n = 78)	(n = 3)	<0.021^2^
1 week	28 (38.4%)	2 (66.7%)	
2 weeks	37 (50.7%)	0 (0%)	
3 weeks	6 (8.2%)	0 (0%)	
4 weeks	2 (2.7%)	0 (0%)	
5 weeks	0 (0%)	1 (33.3%)	
Rifampicin given	(n = 78)	(n = 30)	
Yes	0 (0%)	30 (100%)	
No	78 (100%)	0 (0%)	
Gestational age to start rifampicin (weeks)	-	32.11 ± 3.40	-
Relief in pruritus after rifampicin (n = 30)	-	(n = 30)	1.000^3^
Yes		24 (88.9%)	
No		6 (11.1%)	
Time (in weeks) for relief of pruritus with the addition of rifampicin to UDCA	-	(n = 24)	1.000^3^
1 week		9 (37.5%)	
2 weeks		15 (62.5%)	
Duration of UDCA alone (weeks)***	3.84 ± 2.07	2.86 ± 1.58	0.011^1^
Duration of UDCA + rifampicin (weeks)	-	3.48 ± 1.42	-
Mean ± SD dose of UDCA (mg/day)	911.54 ± 229.05	880.00 ± 260.50	0.563^1^
Mean ± SD dose of rifampicin (mg/day)	-	700.00 ± 363.89	-
Serum bile acids two weeks after drug treatment (umol/L)	22.92 ± 10.67	14.88 ± 10.27	0.080^1^
Predelivery serum bile acids (umol/L)	22.60 ± 7.85	17.25 ± 9.29	0.358^1^

UDCA was started at 32.86 (2.99) weeks in group A and 29.32 (4.24) weeks in group B. Relief in itch was seen in 93.59% (73) in group A and 10% (3) in group B (partial relief). Of those with relief of pruritus in group A (n = 73), 38.4% (28) of the study subjects had a response time of one week, 50.7% (37) had two weeks, 8.2% (6) had three weeks, 2.7% (2) had four weeks, and 0% (0) had five weeks, while in group B (n = 3), two (66.7%) had a response time of one week and one (33.33%) had five weeks. The mean (SD) duration until when the subjects received only UDCA was 3.84 (2.07) weeks and 2.86 (1.58) weeks, respectively, for groups A and B. Rifampicin was added to UDCA in 30 patients belonging to group B, and the mean (SD) gestational age at starting rifampicin was 32.11 (3.4) weeks. The mean duration for subjects in group B receiving UDCA plus rifampicin was 3.48 ± 1.42 weeks. The mean dosage of UDCA given per day was 911.54 ± 229.05 mg in group A and 880.00 ± 260.50 mg in group B. The mean dosage of rifampicin used in group B was 700.00 ± 363.89 mg/day.

The mean (SD) baseline bile acid (pretreatment) was 36.94 (13.00) and 42.50 (15.23) umol/L in groups A and B, respectively (p value = 0.274). At two-week follow-up after treatment, the mean (SD) value of serum bile acids was 22.92 (10.67) and 14.88 (10.27) umol/L in groups A and B, respectively (p value = 0.08).

The fetal outcome for both groups is shown in Table 4. The mean (SD) gestational age at delivery was 37.011 (1.18) weeks in group A and 35.70 (2.57) weeks in group B (p value = 0.012, clinically significant). Of the babies in groups A and B, 63% (46) and 50% (15) were born full term, respectively, and 37% (27) in group A and 50% (15) in group B were born preterm (p value = 0.222). The mean birth weight of babies was 2,706.85 ± 206.19 grams in group A and 2,522.67 ± 342.20 grams in group B (p value = 0.009, significant). There was no significant difference in adverse neonatal outcomes for both groups (68.5% in group A and 70% in group B) (p value = 0.881). In groups A and B, 9% (7) and 6.7% (2) of the patients had an antepartum stillbirth, respectively, and 10.3% (8) of the babies in group A and 6.7% (2) of the babies in group B were born with liquor having dark-colored meconium. In groups A and B, 9% (7) and 6.7% (2) of the babies were born with thin/light green meconium-stained liquor, respectively.

**Table 4:** Neonatal outcome ***Significant at p < 0.05 ^1^t-test ^2^Fisher’s exact test ^3^Chi-squared test NNU: neonatal unit, SB: stillbirth

Neonatal outcome parameters	Group A	Group B	p value
Gestational age at delivery (weeks)***	37.01 ± 1.18	35.70 ± 2.57	0.012^1^
Fetal maturity	(n = 73)	(n = 30)	0.222^3^
Term	46 (63%)	15 (50%)	
Preterm	27 (37%)	15 (50%)	
Spontaneous preterm delivery	13 (17.8%)	8 (26.67%)	0.310
Mean birth weight (grams)***	2,706.85 ± 206.19	2,522.67 ± 342.20	0.009^1^
Adverse perinatal outcome	50 (68.5%)	21 (70%)	0.881^3^
Details of adverse perinatal outcome	(n = 78)	(n = 30)	0.722^2^
None	23 (29.5%)	9 (30%)	
Meconium (thick/dark)/meconium-stained placental membranes/cord	8 (10.3%)	2 (6.7%)	
Antepartum SB	7 (9%)	2 (6.7%)	
Meconium (thin/light)-stained liquor	7 (9%)	2 (6.7%)	
Lost to follow-up	5 (6.4%)	0 (0%)	
NNU admission	28 (35.9%)	15 (50%)	0.182

**Figure 2:**
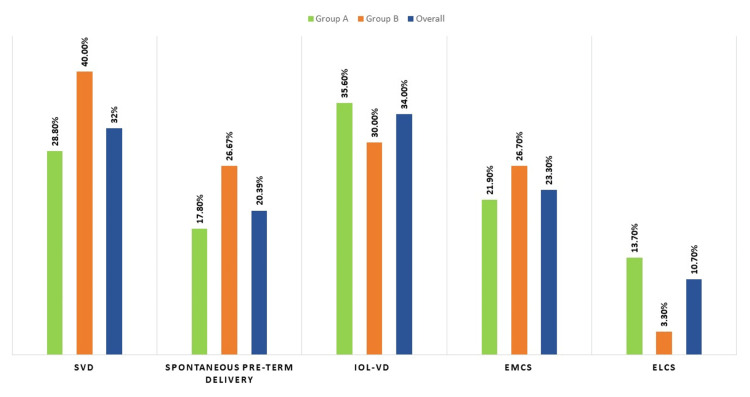
Mode of delivery/birth in both groups and overall study subjects SVD: spontaneous vaginal delivery, spontaneous preterm delivery, IOL-VD: induction of labor followed by vaginal delivery, EMCS: emergency cesarean, ELCS: elective cesarean

Figure 2 shows the mode of delivery in both study groups. There was no significant difference between the two groups in terms of the distribution of the mode of delivery (χ2 = 3.380, p = 0.337). Of the participants in groups A and B and overall study subjects, 28.8%, 40%, and 32% had spontaneous vaginal delivery (SVD). Out of these, 17.8% in group A, 26.67% in group B, and 20.39% in the overall study subjects had spontaneous preterm birth. Induction of labor (IOL) followed by vaginal delivery (VD) was successful in 35.6% of the participants in group A, 30% in group B, and 34% in the overall study subjects. Emergency cesarean (EMCS) was done in 21.9% of the participants in group A, 26.7% in group B (p value = 0.602, non-significant), and 23.3% in the overall study subjects. Of the participants in groups A and B and overall study subjects, 13.7%, 3.3%, and 10.7% underwent elective cesarean (ELCS).

## Discussion

Pruritus is the commonest symptom experienced by patients with ICP. It can significantly impair the quality of life of affected pregnant women, such as inadequate and improper sleep, excoriation of the skin from persistent scratch, and anxiety for their baby in the antenatal period after being counseled for the adverse fetal outcomes that may require iatrogenic preterm and early term deliveries [11]. Its pruritus responds to ursodeoxycholic acid (UDCA). Significant relationships have been found between maternal serum bile acid levels and adverse fetal outcomes. An additional 1%-2% increase in the risk of iatrogenic and spontaneous preterm delivery, stillbirth, and meconium-stained amniotic fluid with a rise of each micromole per liter of serum bile acids above 40 umol/L has been documented [2].

Multiple mechanisms of action of UDCA have been described aiming at one or more of the following pathogenetic processes: protection of injured cholangiocytes against toxic effects of bile acids, stimulation of impaired biliary secretion, and stimulation of detoxification of hydrophobic bile acids, or inhibition of apoptosis of hepatocytes. It is not clear which of these mechanisms plays a primary role in the beneficial therapeutic effects of UDCA [14]. Ursodeoxycholic acid (UDCA) is actually a naturally occurring bile acid, which can displace more hydrophobic endogenous bile salts from the bile acid pool. This leads to the shielding of the hepatocyte membrane from the toxic damage of bile salts and also enhances bile acid clearance across the placenta from the fetus [18]. In vitro studies have shown UDCA to protect rat cardiomyocytes from damage by endogenous bile salts [19].

Despite all the abovementioned benefits of UDCA, not all patients with ICP have symptomatic and biochemical relief (serum bile acids) from UDCA. The PITCHES study, a placebo-controlled randomized controlled trial (RCT) of UDCA in 605 women with ICP of any severity recruited between 20 and 40 + 6 weeks gestation, published that there was a small reduction in pruritus with UDCA, but no benefit in adverse perinatal outcomes [20].

Evidence suggests that rifampicin is an effective second-line drug for controlling pruritus in patients with chronic cholestatic liver disease. It is mostly used as an antipruritic drug in autoimmune cholestatic liver disease and primary biliary cirrhosis (PBC) [14].

Rifampicin is a semisynthetic antibiotic derived from rifamycins, a group of antibacterials produced by *Streptomyces mediterranei*. Rifampicin was initially developed to treat tuberculosis but more recently has been used against other bacteria. The beneficial effect of rifampicin on pruritus was initially discovered serendipitously [21]. It is used extensively despite its broad effects on drug-drug interactions. The molecular mechanism used to explain these broad drug-drug interactions is as follows: rifampicin activates the nuclear pregnane X receptor that in turn affects cytochromes P450 (CYPs), glucuronosyltransferases, and P-glycoprotein activities [22].

CYPs have an important role in protecting organisms from toxic compounds, by bio-transforming lipophilic substrates of diverse structures into more water-soluble metabolites that are then excreted from the body [23,24]. They are essential for eukaryotic life because of their roles in the metabolism of sterols such as cholesterol, bile acids, fatty acids, prostaglandins, leukotrienes, retinoids, and biogenic amines [24,25].

In a questionnaire survey of 27 women affected by ICP, Geenes et al., for the first time, reported about combined UDCA and rifampicin therapy in ICP. After the addition of rifampicin, more than half of the women (54%) had some improvement in serum bile acids, and in 38%, there was a reduction of greater than 50% [26].

The mechanism of action of rifampicin in cholestasis is not very clear, but a study showed that the use of rifampicin in preoperative patients with gallstones led to enhanced bile acid detoxification, bilirubin conjugation, and bilirubin excretion. The same study depicts that these effects were complemented by the upregulation of bile acid export pathways in the liver by UDCA [15]. Therefore, UDCA should not be stopped prior to the commencement of rifampicin because of the complementary effects of both drugs seen in this study. Both drugs should be continued together.

In our study, there was a significant fall in the mean (SD) serum bile acids two weeks after the addition of rifampicin in group B; from 42.50 (15.23) umol/L, it fell to 14.88 (10.27) umol/L. With the combined use of both drugs, the mean ± SD gestational age at delivery was 34 ± 4 weeks (IQR: 33 ± 3 to 35 ± 6 weeks) in the questionnaire study of Geenes et al. [26]. The same was 35.70 ± 2.57 weeks in our prospective observational study.

No adverse effects and additional stillbirths were reported from this combined treatment by Geenes et al. [26]. Our study showed that overall adverse perinatal outcome (neonatal unit admission, meconium staining of placental membranes and cord, meconium-stained liquor, and antepartum stillbirth) was not different in both groups (p value = 0.722) (Table 4). The neonatal unit admission rate for UDCA + rifampicin group was 50% in our study, which is comparable to 46% in the questionnaire study by Geenes et al. The addition of rifampicin did not increase the rate of poor perinatal outcomes, albeit the mean birth weight (p value = 0.009) and gestational age at delivery (p value = 0.012) were less in group B. This is explained by the earlier onset of ICP in group B (p value = 0.001) (Table 3), with a little higher mean serum bile acids than that in group A, and hence, gestational age at delivery was earlier in group B.

For those needing cesarean section in the abovementioned questionnaire study [26], seven (27%) were delivered electively and three (12%) were delivered as an emergency procedure. Contrary to this, in our study, 26.7% of the study subjects in group B (given both UDCA and rifampicin) needed an emergency cesarean and 3.3% underwent planned/elective cesarean.

Due to the limited number of research and the lack of a high level of evidence, an international, multicenter randomized clinical trial to compare the clinical efficacy of rifampicin (300 mg orally twice a day) with that of UDCA tablets (up to 2,000 mg daily) in reducing pruritus in women with ICP using visual pruritus scores as a measuring tool is being carried out presently to examine the outcomes of treatment specifically in the severe early-onset form of ICP [27]. This is going to be the first-time high-quality evidence for the use of rifampicin in severe ICP, which is being conducted in 11 academic hospital centers across Australia and internationally in the UK, Sweden, Finland, and the Netherlands, with over around 108 study participants.

Until the results of this international trial [27] are published, with the available evidence from previous and our study, we support the use of rifampicin in patients who are poor/partial responders to UDCA in terms of pruritus relief and serum bile acid levels. In our study, the median maximum dose of UDCA was approximately 900 mg per day. If there was no/partial response to this dose of UDCA, rifampicin was started from 300 mg per day to a maximum of 900 mg per day, giving a mean value of 700 mg approximately.

## Conclusions

Rifampicin is a good option for those patients with ICP who have partial relief in itch or whose serum bile acids do not fall with UDCA. Nearly 90% (88% in this study) of the patients are relieved of pruritus in 1-2 weeks after adding rifampicin to UDCA. The addition of rifampicin from 300 mg per day and up to a maximum of an average dosage of 700 mg per day does not increase adverse perinatal outcomes (NNU admission, meconium-stained placental membranes/cord, and antepartum stillbirths) compared to that with UDCA alone. Those patients with earlier onset of ICP are more likely to need rifampicin in addition to UDCA for symptomatic and biochemical relief, and due to this early-onset disease, early-term and late-preterm delivery may be required for patients with persistently high levels of serum bile acids and for various indications of fetal distress (non-reassuring fetal heart rate, decreased fetal movements, and passage of meconium). The addition of rifampicin does not make any difference in the mode of delivery when compared to UDCA alone.
